# Emotion-Based Reinforcement Attention Network for Depression Detection on Social Media: Algorithm Development and Validation

**DOI:** 10.2196/37818

**Published:** 2022-08-09

**Authors:** Bin Cui, Jian Wang, Hongfei Lin, Yijia Zhang, Liang Yang, Bo Xu

**Affiliations:** 1 College of Computer Science and Technology Dalian University of Technology Dalian China; 2 College of Information Science and Technology Dalian Maritime University Dalian China

**Keywords:** depression detection, emotional semantic features, social media, sentence-level attention, emotion-based reinforcement

## Abstract

**Background:**

Depression detection has recently received attention in the field of natural language processing. The task aims to detect users with depression based on their historical posts on social media. However, existing studies in this area use the entire historical posts of the users and select depression indicator posts. Moreover, these methods fail to effectively extract deep emotional semantic features or simply concatenate emotional representation. To solve this problem, we propose a model to extract deep emotional semantic features and select depression indicator posts based on the emotional states.

**Objective:**

This study aims to develop an emotion-based reinforcement attention network for depression detection of users on social media.

**Methods:**

The proposed model is composed of 2 components: the emotion extraction network, which is used to capture deep emotional semantic information, and the reinforcement learning (RL) attention network, which is used to select depression indicator posts based on the emotional states. Finally, we concatenated the output of these 2 parts and send them to the classification layer for depression detection.

**Results:**

Experimental results of our model on the multimodal depression data set outperform the state-of-the-art baselines. Specifically, the proposed model achieved accuracy, precision, recall, and F1-score of 90.6%, 91.2%, 89.7%, and 90.4%, respectively.

**Conclusions:**

The proposed model utilizes historical posts of users to effectively identify users’ depression tendencies. The experimental results show that the emotion extraction network and the RL selection layer based on emotional states can effectively improve the accuracy of detection. In addition, sentence-level attention layer can capture core posts.

## Introduction

As an important part of medical informatics research, depression is one of the most dangerous diseases impacting human mental health. It is different from usual mood swings and transient emotional reactions. Long-term depression may cause severe problems for the patient, such as suicide. The World Health Organization (WHO) ranks depression as the most significant cause of disability [1]. Statistics show that over 300 million people suffer from depression all over the world, and the number of patients continues to grow [2]. Depression detection for potential users can help detect the disease at an early stage and help patients get timely treatment.

The latest global digital report [3] shows that there are 4.62 billion social media users worldwide, which is equivalent to 58.4% of the world’s population. Internet users worldwide spend nearly 7 hours a day on the web and 2 hours and 30 minutes on social media. Over the past year, social media users have increased by an average of more than 1 million per day. All these show that social media plays a central role in our daily lives. Meanwhile, an increasing number of people tend to express their emotions and feelings on Weibo, Twitter, etc. People with depression are willing to post depression-related information on social media, such as negative emotions or depression treatment information [4,5]. Therefore, we can obtain a great deal of valuable information about depression from their tweets. The objective of this paper is to predict a label {depression, nondepression} for each user indicating their depressive tendencies by mining their historical posts.

In recent years, psychology-related social media mining has become a research hotspot in natural language processing. The task of detecting users with depression through historical posts on social media has received extensive attention from researchers. Many computer researchers and psychologists have proposed effective methods to detect depression by extracting emotion, interaction, and other features from texts. Nguyen et al [6] extracted emotions, psycholinguistic processes, and content themes in posts to detect users with depression. Shen et al [7] constructed well-labeled depression data sets on Twitter and extracted 6 feature groups associated with depression. Tong et al [8] extracted 3 discriminative features from users’ posts, and then proposed a new cost-sensitive boosting pruning trees model to detect users with depression. Park et al [9] concluded that users with depression prefer to express their status on social media than in real life, so extracting emotional information was essential for depression-detection tasks.

With the maturity of deep learning, the research models have gradually moved from traditional feature engineering to deep learning methods. Yates et al [10] utilized a convolutional neural network (CNN)–based model with multiple inputs for detecting users with depression. Alhanai et al [11] used long short-term memory network (LSTM) to concatenate text and audio representation to detect users with depression. Ren et al [12] extracted emotional information by combining positive words and negative words. Orabi et al [13] investigated the performance differences between recurrent neural network (RNN) models and CNN models in depression detection. Zogan et al [14] fused semantic and user behavior information for detecting depression, and proposed the multimodal depression detection with hierarchical attention network (MDHAN).

**Figure 1:**
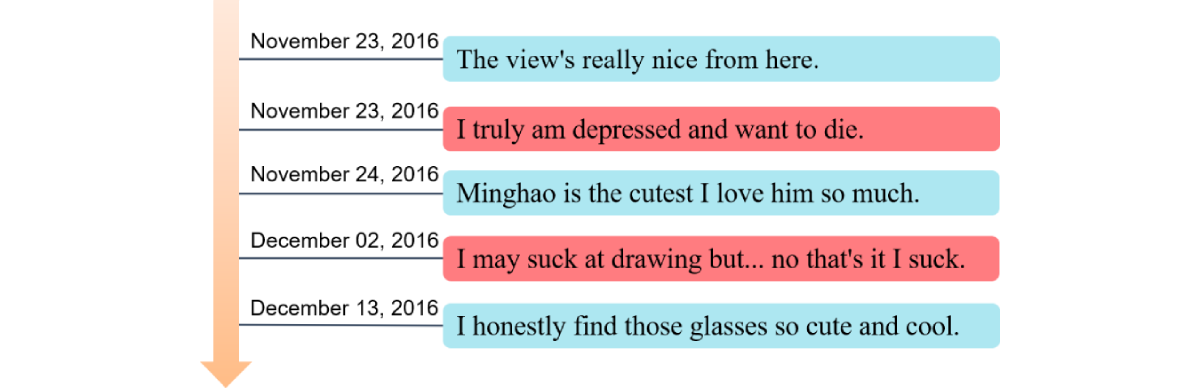
Sample posts of a depressed user. The posts with red highlights are considered depression indicator posts.

All these aforementioned deep learning methods use the entire historical posts of the users. However, it is common for users to share various posts online, and posts related to depression are usually rare. The large number of irrelevant posts contained in historical posts can degrade the performance of the model. Figure 1 illustrates this phenomenon, where posts related to depression are highlighted in red, and the irrelevant posts are highlighted in blue.

From Figure 1, we can see that only a small percentage of tweets are related to depression. Gui et al [15] selected depression indicator posts by reinforcement learning (RL). The advantage of selecting indicator posts is that it excludes the influence of irrelevant posts. If we take all the user’s posts as input, a large amount of noise will be introduced.

From this example, we can also see that there are many emotional words in the user’s posts such as “depressed”, “suck”, “die”, “nice”. However, current methods are lacking in deep mining of emotional information and do not well integrate emotional information into the model. Motivated by these, we propose an emotion-based reinforcement attention network (ERAN) for depression detection in this paper. The proposed model effectively improves the accuracy of depression detection by extracting deep emotional features, selecting depression indicator posts based on the current emotional states, and capturing core information through the sentence-level attention.

The main contributions of this paper can be summarized in the following 3 points:

- First, we extract emotional features by the pretrained TextCNN and fuse the emotional vectors with the output of the attention layer to classify users.
- Second, we improve a reinforcement attention network, which is mainly composed of an RL selection layer and a sentence-level attention layer. The RL selection layer can select depression indicator posts based on the emotional states, and the sentence-level attention captures core information by assigning different weights to posts.
- Finally, experimental results show that the proposed model outperforms the state-of-the-art baselines on the multimodal depression data set (MDD).

## Methods

### Task Definition

Let *H_i_* = {*p*^1^_i_, *p*^2^_i_, ..., *p^T^_i_*} be the set of *T* historical posts of user *u_i_*. The goal of the depression detection is to predict a label 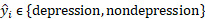 to the user *u_i_* based on historical posts to indicate whether the user is depressed or not.

### Model Overview

**Figure 2:**
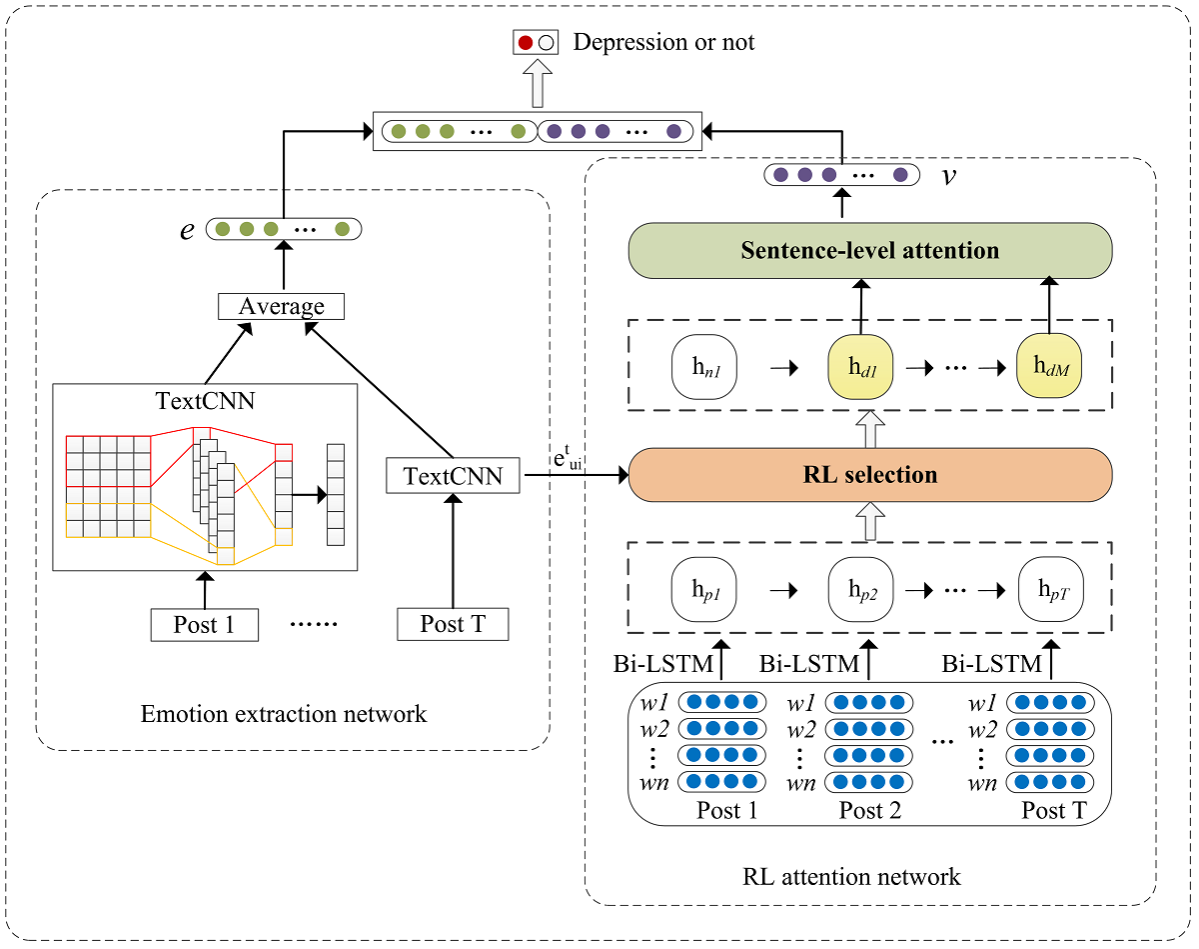
Architecture of the Emotion-Based Reinforcement Attention Network (ERAN). LSTM: long short-term memory network; RL: reinforcement learning.

In the following, we will introduce the structure of our model for depression detection. The proposed model consists of 2 networks, including an emotion extraction network and an RL attention network. The emotion extraction network is used to capture deep emotional sentiment representation from a user’s historical posts. The RL attention network selects depression indicator posts based on the emotional states and assigns weights for the selected posts by the sentence-level attention. Finally, we concatenate the representations captured by the 2 networks and send them to the classification layer to detect whether the user is depressed or not. Figure 2 shows the architecture of the proposed model.

### Emotion Extraction Network

Many studies have shown that emotional information is essential for depression detection on social media. However, current methods fail to extract deep emotional semantic information effectively or do not incorporate the emotional representation well into the model. For instance, some methods just simply concatenate sentiment representation with other information. Motivated by this, we used a pretrained TextCNN [16] to extract deep sentiment features and feed them to the RL attention network of the proposed model to accomplish deep interactions. For user *u_i_*, we input all posts *p^t^_i_* into a pretrained TextCNN. The TextCNN has been pretrained on an emotion classification task labeled as positive, negative, and neutral. After training, the TextCNN is used to extract the emotional information of each post. We regard the last hidden layer vector of the TextCNN as emotion vector 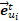. The final emotional semantic representation for all *T*-posts of user *u_i_* is defined as 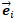, which is the expectation of 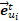:



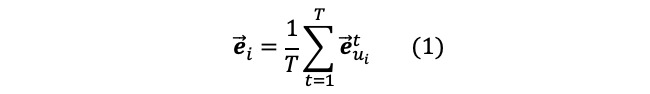



where *T* is the number of posts by user *u_i_* and *t* is *t*th post of the user *u_i_*.

Let 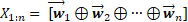 denote the representation of a user’s post, with *n* as the length of the padded post. 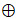 represents the concatenation operator. We utilize word2vec [17] to encode each word *w_i_* as a *d*-dimensional word embedding 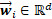.

Then, we input the text sequence *X*_1:_*_n_* into a single-layer CNN. The convolutional layer of the CNN has 3 filters 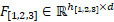. For each 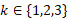, there is *Z* filter *F_k_* for extracting complementary information. And then, we apply them to a window 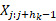 to generate a new feature vector. The feature vector *c_k,j_* is calculated by:



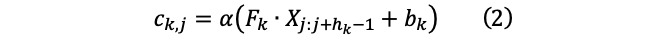



where *α*(·)denotes a nonlinear activation function; 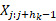 is a window with *h_k_* words, and 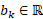 is a bias. For each window in the post {*X*_1:_*_h_*, *X*_2:_*_h_*_+1_, …, *X_n_*_–_*_h_*_+1:_*_n_*}, the above actions are taken to get a feature map 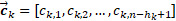, where 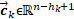, and *h_k_* is the height of the convolution kernel.

After convolution operation, each filter *F_k_* creates *Z* feature maps 
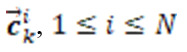
. Following this, to extract the maximum features, we connect a max-pooling operation [18] to all feature maps. The output is calculated as 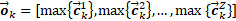. The output of max-pooling, which covers all feature maps 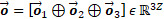, is the concatenation of each ***o****_k_*. Finally, 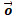 is entered into a fully connected layer. The output of the classification layer is calculated as:



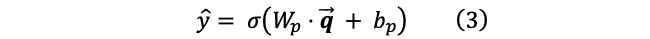



where 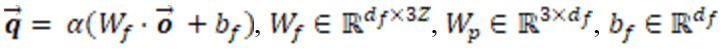, and 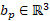; *α*(·) is a nonlinear activation function. The fully connected layer is followed by a sigmoid-classification layer with 3 classes, and *σ*(·) represents sigmoid operation.

### RL Attention Network

#### Overview

Users’ historical posts usually contain various content, and only a small fraction may be related to depression. Those irrelevant posts pose a challenge to identify users’ depressive tendencies effectively, so we need to develop a model to select only depression-related posts. The historical posts of the user *u_i_* are denoted as *H_i_* = {*p*^1^*_i_*, *p*^2^*_i_*, …, *p^T^_i_*}, and the depression indicator posts are denoted as 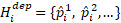.

The structure of this network includes (1) a bidirectional LSTM (BiLSTM) that generates contextual representation, (2) an RL selection layer that chooses depression-related posts based on the current emotional states from *H_i_*, and (3) a sentence-level attention layer that allows the model to pay more attention to higher-weight posts.

#### BiLSTM Layer

Graves et al [19] proposed the BiLSTM, which has been widely used in natural language processing to capture long-distance contextual dependency. Superior to LSTM [20], BiLSTM can capture bidirectional semantic dependencies. Inspired by this, we utilized BiLSTM to encode contextual information. The algorithm processes of LSTM are as follows:

*f_k_* = *σ*(*W^f^*·[*h_k_*_–1_, *x_k_*] + *b^f^*) (**4**)

*i_k_* = *σ*(*W^i^*·[*h_k_*_–1_, *x_k_*] + *b^i^*) (**5**)

*o_k_* = *σ*(*W^o^*·[*h_k_*_–1_, *x_k_*] + *b^o^*) (**6**)

*c_k_* = tanh(*W^c^*·[*h_k_*_–1_, *x_k_*] + *b^c^*) (**7**)

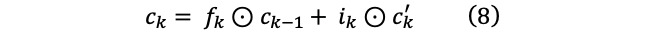





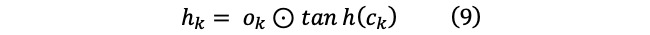



where *W^f^*, *W^i^*, *W^o^*, and *W^c^* are parameters that can be trained. 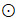 represents the element-wise multiplication operation, *x_k_* denotes the pretrained word2vec embedding, and *σ*(·) represents sigmoid function.

Given an input sequence *X* = [*x*_1_, *x*_2_, ..., *x_n_*], the forward hidden state is 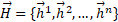, and the backward hidden state is 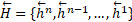. The representation of the sentence is:



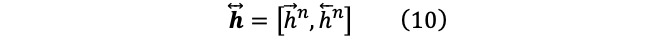



For user *u_i_*, the representation of posts is 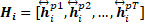, where *T* is the number of posts.

#### RL Selection Layer

Because we only have user-level labels, it becomes a key challenge to select posts related to depression. Gui et al [15] utilized RL to select depression indicator posts. However, their method still has a high recognition accuracy in the unselected posts, which indicates that this model misses many important posts. Inspired by this, we introduced emotional states to improve the selection strategy based on RL.

RL is a way of learning by “trial and error” in the environment. It has 3 important factors: agent, environment, and reward, where the agent is the selector. At each step *t*, the agent executes the action *a^t^* based on the state *s^t^* to select the current post or not. After executing all posts, the classifier gives the agent a total reward to evaluate the performance of this policy. Policy gradient [21] is an optimization method of parameterizing the policy, which optimizes the parameter *θ* to maximize the total reward. Next, we will explain these parts.

In this layer, after encoding, the post *p^t^* is denoted by the vector 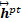. At each step *t*, the current post is 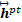, the selected posts set is 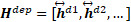, and the unselected posts set is 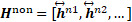. If action *a^t^*=1, the post 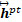 is appended to 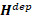; otherwise 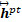 is appended to ***H***^non^, where 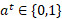. The state *s^t^* with emotional vector is represented as follows:



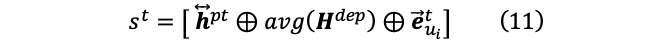



where 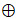 represents the concatenation operation, and avg(·) represents the average operation. 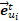 denotes the emotion vector of the *t*th post of *u_i_*. The current state *s^t^*incorporates the emotion vector, which enables the agent to take better actions. The action obeys the following policy to take actions:

π(*a^t^|s^t^*; *θ*) = *p_θ_*(*a^t^|s^t^*, *θ*) **(12)**

where *θ* represents the parameter of the policy function and is optimized to maximize the total reward, (*a^t^|s^t^* ;*θ*) represents the policy function that the agent follows to take action, and *p_θ_* (*a^t^|s^t^*, *θ*) is a probability distribution over the action, and we serialize the discrete policy via the *MLP* layer.

For each episode *τ* = {*s*^1^, *a*^1^, *s*^2^, *a*^2^, ..., *s^T^*, *a^T^*, END} of user *u_i_*, the classifier will return a reward after all the selections are made. The objective is to maximize the reward of the episode. The reward is defined as the predicted probability after executing this episode:

*R*(*τ*) = *p*(*y_i_*|*H*^dep^; *θ*′) (**13**)

where *θ*′ represents the parameters of the classification layer and is optimized by the depression classifier.

After *N* sampling for user *u_i_*, we get *N* episodes *τ* = {*τ*_1_, ..., *τ*_N_}. To optimize the parameter *θ*, we calculate the expectation of *R*(*τ*). The calculation processes are as follows:



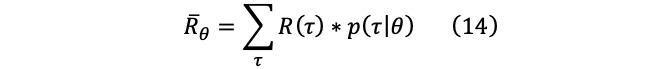





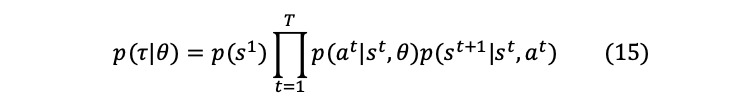



Here, because the transfer between states is Markovian, we will use the chain rule to calculate *p*(*τ*|*θ*), as shown in Equation (15).

To maximize 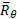, we calculate its gradient against *θ*. The equation is shown as follows:



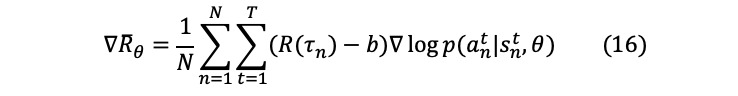



Here, to simplify the objective function, we assume that the probability of each occurring is 1/*N*. In the equation, 
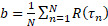
 is a baseline value. If *R*(*τ_n_*) – *b* is positive, the optimization will proceed toward increasing the probability *p*(*a^t^*|*s^t^*, *θ*). If *R*(*τ_n_*) – *b* is negative, the optimization will proceed toward reducing the probability. Thus, is updated in this way: 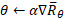, where *α* is the learning rate.

Finally, the loss function of this part is calculated by:



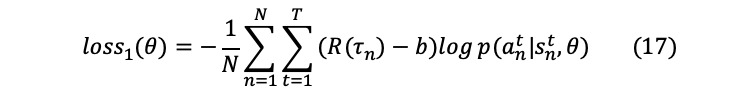



Here, maximizing *R*(*τ*) is minimizing loss_1_(*θ*) actually. The parameters, as well as the loss, will be optimized by the gradient. After the selection of agent, 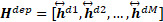 contains the posts related to depression. Then we feed ***H****^dep^* into the attention layer.

#### The Sentence-Level Attention Layer

The semantics of a document can be described by a few sentences in the document. The model will not capture the key information if it treats each sentence fairly. To solve the document classification problem, Yang et al [22] designed the hierarchical attention network. This network contains a word-level attention used to focus on keywords and a sentence-level attention used to focus on critical sentence. Inspired by this, we utilized the sentence-level attention mechanism to enable our model to focus on relevant posts. It will create an attention weight for each post in ***H****^dep^*, and the model will focus more on tweets with higher weights.

We assume that the depression indicator posts set of *u_i_* is 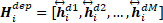, which has *M* indicator posts after padding. For the vector 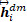, the attention weight is calculated by:



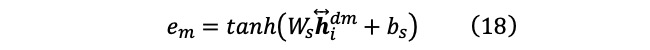





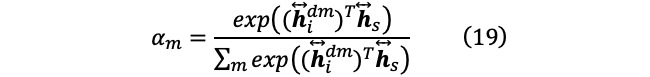





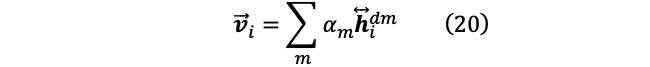



where 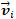 is the final posts representation that summarizes all the posts in ***H****_i_^dep^* 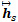 is a vector used to measure the weight of the posts and is randomly initialized. During the training process, 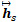 can be updated.

### Final Prediction

In the classifier, we concatenate the output of attention layer 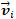 and emotion representation 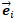 to form the unified text representation 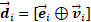. Finally, 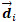 is projected to the output layer having 2 neurons with a soft-max activation. The categorical cross-entropy loss function and the soft-max probability are calculated as follows:



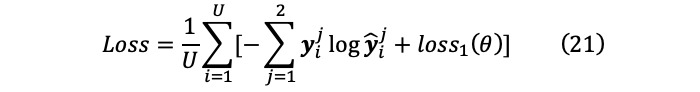





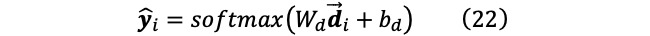



where, *j* represents the categories, *U* is the total number of users in data set, 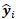 represents the classification probability, and ***y****_i_^j^* is the ground truth.

### Ethics Approval

The data set and methods used in this work are publicly available and do not involve any ethical or moral issues.

## Results

### Data Sets

Shen et al [7] proposed the MDD data sets, which contain well-labeled data sets *D*_1_, *D*_2_, and an unlabeled data set *D*_3_ on Twitter. These 3 data sets collect posts from users on Twitter at specific times. Table 1 describes the statistics of these 3 data sets, including the number of users and tweets.

- Depression data set *D1*: Based on the tweets between 2009 and 2016, if users’ tweets satisfy the strict pattern “(I’m/ I was/ I am/ I’ve been) diagnosed depression,” they will be labeled as depressed.
- Nondepressed data set *D2*: In this data set, only users who have never posted tweets containing “depress” are marked as nondepressed.
- Depression-candidate data set *D3*: In this data set, users are obtained if their anchor tweets loosely contain “depress.” In this way, *D3* contains more users with depression than randomly sampling.

In our experiments, we added all the users in *D*_1_ to the data set. In addition, we randomly selected the same number of users in *D*_2_ to balance the data set. Selection rules excluded users with less than 15 posts, or users with non-English posts. The data set used in this paper contained 2804 Twitter users and over 500,000 posts made by them. Finally, we used 2243/2804 (79.99%) users in the data set to train our model and 561/2804 (20%) users to test our model.

**Table 1:** Summary of the data sets.

Data set	Label	User	Tweets
*D*_1_	Depressed	1402	292,564
*D*_2_	Nondepressed	>300 million	>10 million
*D*_3_	Nonlabeled	36,993	35,076,677

### Evaluation Metrics

In the experimental phase, we used accuracy, precision, recall, and *F*_1_-score to evaluate the performance of the proposed model. *F*_1_-score is calculated as follows:

*F*_1_ = (2·*P*·*R*)/(*P* + *R*) (**23**)

where *R* = *TP*/(*TP*+*FN*) and *P* = *TP*/(*TP*+*FP*); here, *P* is precision, *R* represents recall, *TP* represents true-positive prediction, *FN* is false-negative prediction, and *FP* is false-positive prediction.

### Experimental Setting

During the experimental phase, the hyperparameters were randomly initialized based on our experience. The pretrained word2vec is used to initialize the word embeddings. The Adam optimizer [23] was used to optimize the hyperparameters. Other hyperparameter settings are shown in Table 2.

The training of our ERAN model is based on the operating system of Ubuntu 18.04, using PyTorch version 1.9.0 and Python version 3.7.0. The graphics processing unit is NVIDIA TITAN Xp with 12-GB memory.

**Table 2:** Values of hyperparameters.

Hyperparameters	Value
Word embedding dimension	300
BiLSTM^a^ hidden units	200
Dropout rate	0.5
Batch size	128
Learning rate	0.001

^a^BiLSTM: bidirectional long short-term memory network.

### Comparison With Existing Methods

Here, we describe the baseline methods that we compared with.

- Naïve Bayesian (NB): NB [24] is widely used in classification tasks. The classifier accepts all features to detect the user’s depressive tendencies.
- Wasserstein Dictionary Learning (WDL): Rolet et al [25] proposed the WDL. It considers the Wasserstein distance as the fitting error to leverage the similarity shared by the features.
- Multiple Social Networking Learning (MSNL): Song et al [26] proposed the MSNL model to solve the volunteerism tendency prediction problem.
- Multimodal Depressive Dictionary Learning (MDL): Shen et al [7] proposed the MDL model by combining the multimodal strategy and dictionary learning strategy.
- CNN/LSTM + RL: Gui et al [15] proposed an RL model to select depression indicator posts.
- MDHAN: Zogan et al [14] proposed MDHAN. They extracted semantic information using a hierarchical attention network and user behavior by a multimodal encoder.

We compared the performance of the proposed model (ERAN) with other existing models on the MDD data set. The experimental results are shown in Table 3.

From the first 4 classic methods, MDL achieves the best performance with 78.6% in *F*_1_-score, indicating the validity of the multimodal depressive dictionary. The results based on BiLSTM are better than those based on LSTM, indicating that the bidirectional encoder can capture more helpful information. Similarly, the performances based on BiLSTM (Att) are better than those based on BiLSTM, which can demonstrate that the sentence-level attention mechanism can capture more important depression information.

With the popularity of pretrained approaches, we experimented with 2 pretrained models, Bidirectional Encoder Representations from Transformers (BERT) and Robustly Optimized BERT pre-training Approach (RoBERTa) [27], and fine-tuned them on our data set. From Table 3, we can see that the simple pretraining models do not work very well, which may be due to the sparse distribution of depression-related words causing the pretrained models to fail to maximize their ability.

**Table 3:** Results compared with the baseline models.

Model	Accuracy	Precision	Recall	*F*_1_-score
NB^a^ [22]	0.636	0.724	0.623	0.588
WDL^b^ [24]	0.761	0.763	0.762	0.762
MSNL^c^ [25]	0.782	0.781	0.781	0.781
MDL^d^ [6]	0.790	0.786	0.786	0.786
LSTM^e^	0.797	0.812	0.813	0.812
BiLSTM^f^	0.805	0.817	0.818	0.817
BiLSTM (Att^g^)	0.817	0.828	0.828	0.828
BERT^h^ (base) [27]	0.845	0.883	0.825	0.853
RoBERTa^i^ (base) [27]	0.851	0.902	0.837	0.868
CNN^j^ + RL^k^ [14]	0.871	0.871	0.871	0.871
LSTM + RL [14]	0.870	0.872	0.870	0.871
MDHAN^l^ [13]	0.895	0.902	0.892	0.893
ERAN^m^ (ours)	0.906	0.912	0.897	0.904

^a^NB: naïve Bayesian.

^b^WDL: Wasserstein Dictionary Learning.

^c^MSNL: Multiple Social Networking Learning.

^d^MDL: Multimodal Depressive Dictionary Learning.

^e^LSTM: long short-term memory network.

^f^BiLSTM: bidirectional long short-term memory network.

^g^Att: attention.

^h^BERT: Bidirectional Encoder Representation from Transformers.

^i^RoBERTa: Robustly Optimized BERT pre-training Approach.

^j^CNN: convolutional neural network.

^k^RL: reinforcement learning.

^l^MDHAN: multimodal depression detection with hierarchical attention network.

^m^ERAN: emotion-based reinforcement attention network.

The CNN/LSTM + RL models use RL to select indicator posts, which verifies the validity of the selection strategy. The MDHAN model proves that the multimodal features are also important by fusing semantic information with user behavior information.

The proposed ERAN model achieves optimal results because we fused emotional information and selected depression indicator posts based on emotional states. In addition, the sentence-level attention can capture core posts.

### Ablation Study

**Figure 3:**
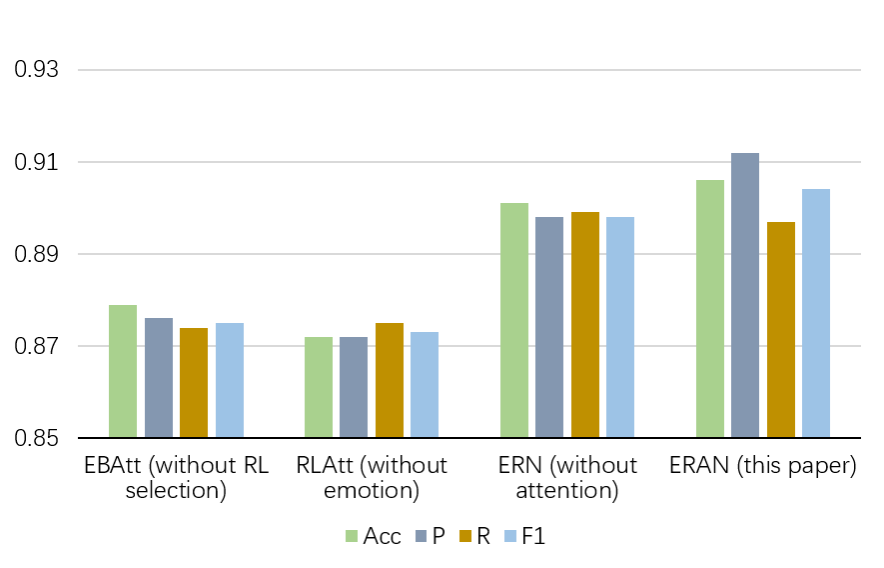
Results of ablation experiments. Emotion-Based Reinforcement Attention Network (ERAN) is the proposed model, and the remaining three are the models after removing one module of ERAN. Acc: accuracy; EBatt: emotion-based BiLSTM (bidirectional long short-term memory network) attention network; ERN: emotion-based reinforcement learning network; F1: *F*_1_-score; P: precision; R: recall; RLAtt: reinforcement learning attention;

Ablation experiments were conducted to validate the necessity of the emotion extraction network, the RL selection layer, and the sentence-level attention. The study is performed by removing one module at a time. The results of the ablation experiments are presented in Figure 3.

Emotion-based BiLSTM attention network (EBAtt) is the model that removes the RL selection layer from the proposed model and uses all user posts. Reinforcement learning attention network (RLAtt) is the model that removes the emotion extraction network. Emotion-based reinforcement learning network (ERN) is the model that substitutes the sentence-level attention with the averaging operation. We can see that the ERAN model proposed in this paper performs best. Although ERAN is lower than ERN in precision, it is higher in the other 3 metrics. The sentence-level attention can improve the performance, demonstrating that it can capture more important posts.

EBAtt extracts semantic information on all posts by BiLSTM and fuses it with emotional representation. Results show that the *F*_1_-score of EBAtt decreases by 2.9% compared with the proposed model, which indicates the necessity of selecting depression indicator posts.

RLAtt is the model after removing the emotion extraction network from ERAN. Similarly, the state of the RL selection layer does not contain the emotion vector. The *F*_1_-score of RLAtt is lower than the proposed model by 3.1%, which indicates that the emotional information improves our model the most.

From the results, we can conclude that extracting emotional information through the pretrained TextCNN is beneficial for depression detection task. Selecting depression indicator posts based on emotional states is also necessary for depression detection. In addition, the sentence-level attention layer can focus on useful posts.

### The Effectiveness of The RL Selection Layer

**Figure 4:**
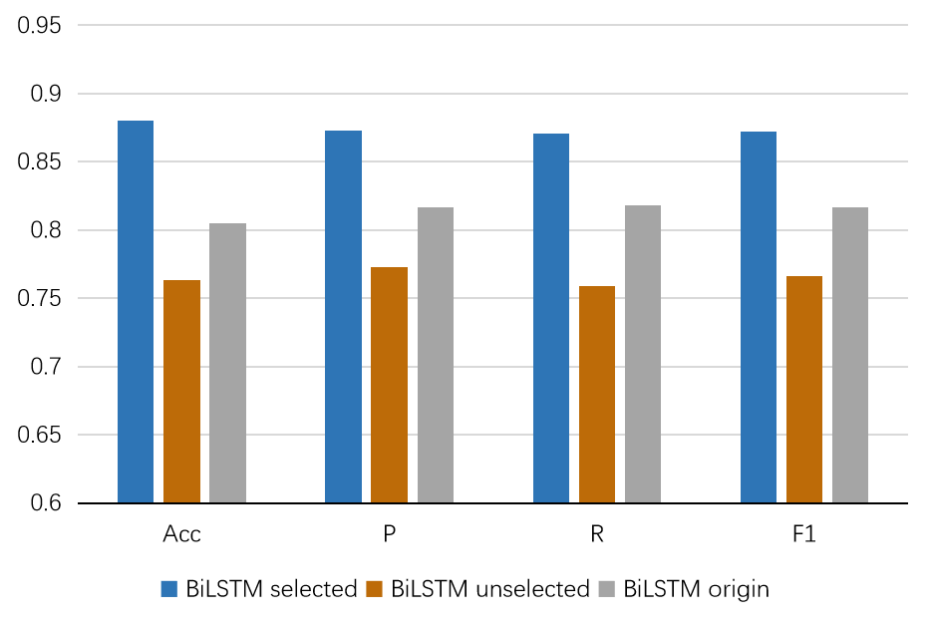
Comparative results of BiLSTM trained on the selected posts, the unselected posts, and the original posts. Acc: accuracy; BiLSTM: bidirectional long short-term memory network; F1: *F*_1_-score; P: precision; R: recall.

We train the proposed model to generate 2 subsets of depression-related and unselected posts from the original data set. Following this, we obtain 3 data sets, the selected indicator data set *H^dep^*, the unselected data set *H^non^*, and the original data set *H^orig^*. The baseline model BiLSTM is then trained on each of these 3 data sets to verify the effectiveness of the RL selection layer. Figure 4 illustrates the results of the baseline model BiLSTM on the 3 data sets.

From Figure 4, we can conclude that the model trained on *H^dep^* performs best. Meanwhile, the model trained on *H^non^* achieves worse performance than the one trained on *H^orig^*, which demonstrates the effectiveness of the RL selection.

To verify the effectiveness of introducing sentiment vectors in the RL selection module, we removed the sentiment vector 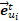 in the state *s^t^*. The ablation experiment achieves 88.3%, 88.1%, 87.3%, and 87.7% in accuracy, precision, recall, and *F*_1_-scores, respectively. Through the results of the ablation experiment, we can find that the performance of the model decreases after removing the sentiment vectors from the RL selection module, which proves that the sentiment information is helpful for selecting depression indicator posts.

### Attention Visualization and Error Analysis

**Figure 5:**
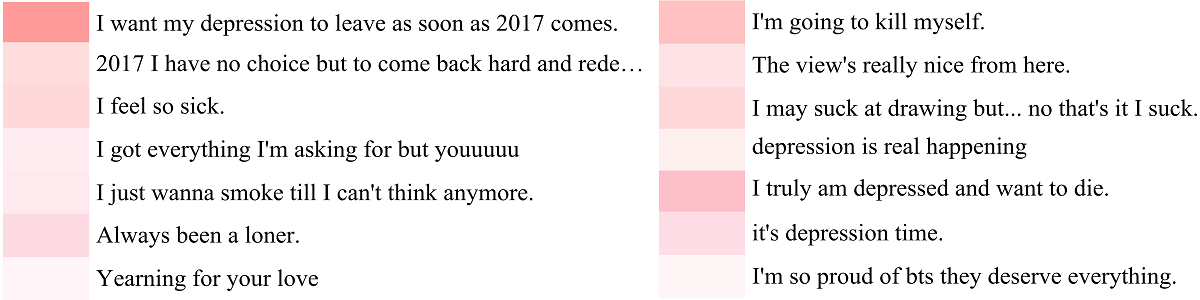
Examples of attention visualization. Different colors represent different weights. The deeper the color, the greater the weight of the post.

In this section, we extracted attention weights and visualized them to verify the validity of the sentence-level attention layer and the reasonableness of the selected posts. We have selected a part of the results of the users as examples, who are called “___mandyy” and “Adri.” The results of attention visualization are illustrated in Figure 5.

The first example shows that the first post has the highest weight, where “my depression” indicates that the user has depression. The second post also contains the words “depression”, “me”, etc. Thus, “___mandyy” is finally classified as having “depression.” As we can see, many of the selected posts of this user with depression are of negative sentiment, suggesting a strong association between depression and negative emotions.

The second user is the one we have used as an example in Figure 1. From the results of the visualization, we can observe that the fifth post has the highest weight. Classification results indicate that the user is indeed depressed. However, the posts “The view’s really nice from here.” and “I’m so proud of bts they deserve everything” are irrelevant to depression. In addition, the model assigns high weight to the first irrelevant post. One possible reason for choosing these posts is that they contain strong emotional expressions. We think it can be improved by developing a stricter selection strategy.

## Discussion

### Principal Findings

Based on the results, we can observe that introducing emotional information can be very helpful for depression detection tasks, indicating that emotional characteristics are strongly associated with depression. The strategy of selecting depression indicator posts from historical posts is critical to our model because it excludes the effect of irrelevant information. As only user-level labels are in the data set, we use RL to select posts rather than supervised learning. Furthermore, the fusion of emotion vectors into agent states is interpretable. The sentence-level attention layer assigns greater weight to relevant posts, which makes the model perform better.

Although the RL selection layer performs well, the selected posts still contain irrelevant posts with strong emotional expressions. Compared with other optimization methods, the convergence of policy gradient is better. However, this method tends to fall into local optimum and its training speed is slow.

### Conclusions

In this paper, we addressed the task of depression detection of users on social media by proposing an ERAN. The proposed model contains 2 modules: the emotion extraction network and the RL attention network. It uses the pretrained word2vec embeddings as input. The emotion extraction network captures deep emotional information by a pretrained TextCNN. The RL attention network is composed of the BiLSTM layer, the RL selection layer, and the sentence-level attention layer. The RL selection layer can select depression indicator posts from original posts based on the emotional states, and the attention layer is able to assign greater weight to relevant posts. Results show that the proposed model outperforms the state-of-the-art model. We verified the validity of the emotion extraction network, the RL selection layer, and the sentence-level attention layer through an ablation study and a visualization analysis. The emotional features and selection of indicator posts are necessary for depression detection task.

The proposed model uses social media data set to detect depression, which can provide a certain degree of diagnostic basis and address the problem of the lack of effective objective diagnosis in the field of depression. In the future work, we will introduce users’ personality information and multimodal information such as visual information to our model. We will further extract more detailed information about depression based on the proposed model to help analyze the pathogenesis of depression as well as accurate treatment.

## Abbreviations

BERT: Bidirectional Encoder Representations from Transformers
BiLSTM: bidirectional long short-term memory network
CNN: convolutional neural network
EBAtt: emotion-based BiLSTM attention network
ERAN: emotion-based reinforcement attention network
ERN: emotion-based reinforcement learning network
LSTM: long short-term memory network
MDHAN: multimodal depression detection with hierarchical attention network
MDD: multimodal depression data set
MDL: Multimodal Depressive Dictionary Learning
MSNL: Multiple Social Networking Learning
NB: naïve Bayesian
RL: reinforcement learning
RLAtt: reinforcement learning attention
RNN: recurrent neural network
RoBERTa: Robustly Optimized BERT pre-training Approach
WDL: Wasserstein Dictionary Learning
WHO: World Health Organization
